# Discovery and characterization of ionic liquid-tolerant thermophilic cellulases from a switchgrass-adapted microbial community

**DOI:** 10.1186/1754-6834-7-15

**Published:** 2014-01-29

**Authors:** John M Gladden, Joshua I Park, Jessica Bergmann, Vimalier Reyes-Ortiz, Patrik D’haeseleer, Betania F Quirino, Kenneth L Sale, Blake A Simmons, Steven W Singer

**Affiliations:** 1 Physical Biosciences Division, Lawrence Berkeley National Laboratory, Joint BioEnergy Institute (JBEI), 1 Cyclotron Road, Berkeley, CA 94720, USA; 2 Biological and Materials Science Center, Sandia National Laboratories, Livermore, CA, USA; 3 Physical and Life Sciences Directorate, Lawrence Livermore National Laboratory, Livermore, CA, USA; 4 Department of Geochemistry & Department of Ecology, Earth Sciences Division, Lawrence Berkeley National Laboratory, Berkeley, CA, USA; 5 Department of Genomics Science and Biotechnology, Universidade Católica de Brasília, Brasília DF 70790-160, Brazil; 6 Embrapa-Agroenergy, Brasilia DF 70770-901, Brazil; 7 Current address: Department of Biological Sciences, Takeda California, Inc., San Diego, CA, USA

**Keywords:** Cellulase, Ionic liquid, Thermophilic, Biofuel

## Abstract

**Background:**

The development of advanced biofuels from lignocellulosic biomass will require the use of both efficient pretreatment methods and new biomass-deconstructing enzyme cocktails to generate sugars from lignocellulosic substrates. Certain ionic liquids (ILs) have emerged as a promising class of compounds for biomass pretreatment and have been demonstrated to reduce the recalcitrance of biomass for enzymatic hydrolysis. However, current commercial cellulase cocktails are strongly inhibited by most of the ILs that are effective biomass pretreatment solvents. Fortunately, recent research has shown that IL-tolerant cocktails can be formulated and are functional on lignocellulosic biomass. This study sought to expand the list of known IL-tolerant cellulases to further enable IL-tolerant cocktail development by developing a combined in vitro/in vivo screening pipeline for metagenome-derived genes.

**Results:**

Thirty-seven predicted cellulases derived from a thermophilic switchgrass-adapted microbial community were screened in this study. Eighteen of the twenty-one enzymes that expressed well in *E. coli* were active in the presence of the IL 1-ethyl-3-methylimidazolium acetate ([C_2_mim][OAc]) concentrations of at least 10% (v/v), with several retaining activity in the presence of 40% (v/v), which is currently the highest reported tolerance to [C_2_mim][OAc] for any cellulase. In addition, the optimum temperatures of the enzymes ranged from 45 to 95°C and the pH optimum ranged from 5.5 to 7.5, indicating these enzymes can be used to construct cellulase cocktails that function under a broad range of temperature, pH and IL concentrations.

**Conclusions:**

This study characterized in detail twenty-one cellulose-degrading enzymes derived from a thermophilic microbial community and found that 70% of them were [C_2_mim][OAc]-tolerant. A comparison of optimum temperature and [C_2_mim][OAc]-tolerance demonstrates that a positive correlation exists between these properties for those enzymes with a optimum temperature >70°C, further strengthening the link between thermotolerance and IL-tolerance for lignocelluolytic glycoside hydrolases.

## Background

With global energy demands rising rapidly, new technologies need to be developed that utilize new resources for transportation fuels. Lignocellulosic biomass is one promising resource, where an estimated one billion tons will be available annually by 2030 in the US alone [1]. Lignocellulosic biomass is primarily composed of plant cell-wall polysaccharides, such as cellulose and hemicelluloses, which together constitute 60 to 70% of the biomass by weight for potential energy crops such as switchgrass [2]. These polymers are composed of hexose and pentose sugars that can be fermented into substitutes for gasoline, diesel and jet fuel [3-7], augmenting or partially displacing current petroleum-based sources of liquid transportation fuels. One of the challenges of using lignocellulosic biomass for production of biofuels is the recalcitrance of plant biomass to deconstruction, a property that necessitates some form of chemical or physical pretreatment to permit enzymes or chemicals to gain access to and hydrolyze the plant polymers into fermentable sugars [4,6,8]. This study focuses on this challenge and discloses the discovery and characterization of biomass-deconstructing enzymes that are more compatible with certain forms of biomass pretreatment solvents than the current commercially available enzyme cocktails.

The recalcitrance of lignocellulosic biomass has been a difficult hurdle to overcome, but promising new technologies using certain ionic liquids (ILs) that have come about in the last decade indicate that we are well on our way to moving past this barrier [9,10]. Pretreating biomass with certain classes of ILs, most notably those with imidazolium-based cations, can be more efficient and tunable than other existing forms of pretreatment, and technoeconomic analysis of IL-pretreatment suggests that there are potential routes to economic viability [8,11]. One remaining issue with this technology that needs to be addressed to maximize efficiency and reduce capital costs is the incompatibility of ILs with cellulase cocktails derived from filamentous fungi. These enzyme cocktails can be strongly inhibited by certain ILs, such as 1-ethyl-3 methylimidazolium acetate [C2mim][OAc], necessitating expensive and inefficient washing steps to remove residual IL from the biomass prior to addition of enzymes [8,12-14]. One solution to this issue is to develop enzyme cocktails that are tolerant to ILs. Fortunately, it has been shown that certain thermophilic bacterial cellulase enzymes can tolerate high levels of the IL [C2mim][OA]], and in fact these enzymes have been used to develop an IL-tolerant cellulase cocktail called JTherm [13-17]. It has been further demonstrated that JTherm can be used in a one-pot IL pretreatment and saccharification bioprocessing scheme that eliminates the need to wash the pretreated biomass with water, significantly reducing the number of process steps [18].

The next step toward an economically viable IL-based bioprocessing scheme for the conversion of lignocellulosic biomass to biofuels will be to further integrate and improve all components of the process. For IL-tolerant cellulase cocktails, this includes reducing enzyme loadings by reformulating the cocktails to achieve greater saccharification efficiency; a modification predicted by technoeconomic modeling to substantially reduce overall costs in a biorefinery [11]. Enzyme cocktail reformulation will require screening through an expansive list of IL-tolerant cellulase enzymes to identify those that enhance saccharification efficiency under conditions likely to be found in a biorefinery. However, the number of known IL-tolerant cellulase enzymes, specifically those tolerant to [C2mim][OAc], is quite small, an issue that hampers cocktail reformulation efforts. The goal of this study was therefore to discover and characterize an expanded set of [C2mim][OAc]-tolerant cellulase enzymes to enable future development of highly efficient IL-tolerant biomass-deconstructing enzyme cocktails. This study focused on thermophilic organisms based on clues provided by previous studies that indicate that thermotolerance may be positively correlated with IL-tolerance. Hence, we can leverage a naturally evolved physiological characteristic of an enzyme and use it as a proxy to discover enzymes with a non-natural industrially relevant characteristic, such as IL-tolerance.

This concept drove recent work where complex compost-derived microbial communities were cultivated on switchgrass under thermophilic conditions to enrich for organisms that produce mixtures of IL-tolerant cellulases and xylanases [14]. The community was composed of several abundant bacterial populations related to *Thermus thermophilus, Rhodothermus marinus, Paenibacillus*, *Thermobacillus* and an uncultivated lineage in the *Gemmatimonadetes* phylum [19]. The glycoside hydrolases from this community were found to have high optimum temperatures (approximately 80°C) and tolerated relatively high levels of [C2mim][OAc] compared to commercial cellulase cocktails (>50% activity in the presence of 30% (v/v) [C2mim][OAc]). Therefore, these communities provide a rich reservoir of potential enzyme targets to develop thermophilic and IL-tolerant cellulase cocktails to be used in lignocellulosic biofuel production platforms. To discover the genes that encode these IL- and thermo-tolerant enzymes, metagenomic and proteomic analysis was conducted on the community [14,19]. The analysis identified a variety of genes encoding potential cellulose and hemicellulose-degrading enzymes, a subset of which were assembled into complete open reading frames (ORFs) from the metagenome. To validate the concept that thermotolerance can be used as an engine for discovery of IL-tolerant enzymes, this study expressed and characterized 37 of these predicted cellulase genes from the metagenome using both cell-free and *in vivo Escherichia coli* (*E. coli*) expression systems. Both expression methods were employed to determine which method is most suitable for rapid and efficient screening of metagenome-derived gene sets. We found that several of the ORFs encode IL- and thermo-tolerant cellulase enzymes, including enzymes with activities that are stimulated in the presence of ILs.

## Results

### Identification of cellulases in a switchgrass-adapted metagenome

The metagenome of a thermophilic switchgrass-degrading bacterial community was curated for genes with cellulase-related annotations or homology to sequences for cellulase enzymes deposited in the CAZy database (http://www.cazy.org/), including β-glucosidases (BG), cellobiohydrolases (CBH), and endoglucanases (Endo). A total of nineteen predicted BGs, two CBHs, and sixteen Endos were identified that appeared to be complete ORFs (Table 1; see Methods). The top BLASTP hit for each identified cellulase is indicated in Table 1, including the maximum identity and source organism of the top hit in GenBank. Many of the ORFs are homologous to those found in isolates that cluster with abundant community members, such as *Rhodothermus marinus, Paenibacillus*, *Thermobacillus* and *Gemmatimonadetes*. Many of the ORFs fall into sequence bins assigned to these organisms in the metagenome that are consistent with the phylogenetic affiliation predicted by the BLASTP search (Table 1, Additional file 1, and D’Haeseleer *et. al*. 2013 [19]). Several of the ORFs in Table 1 contained sequencing errors or were identified as fragments and were manually corrected/assembled (see Methods for details). For J08/09 and J38/39, the manual assembly resulted in two closely related proteins, and therefore both versions were tested.

**Table 1:** Predicted cellulase enzymes identified in the switchgrass-adapted metagenome

**Gene ID**	**IMG gene ID**	**GH family**	**Predicted function**	**Max identity (%)**	**Genbank accession number**	**Top Blast-hit organism**	Metagenome bin*
*J01*	2061974227	3	β-glucosidase	42	ZP_06970881.1	Ktedonobacter racemifer DSM 44963	Paenibacillus
*J02*	2061976655	3	β-glucosidase	97	YP_003321925.1	Thermobaculum terrenum	Thermobaculum
*J03*	2061976732	3	β-glucosidase	96	YP_003322827.1	Thermobaculum terrenum	Thermobaculum
*J04*	2061977694	1	β-glucosidase	62	ZP_10205923.1	Rhodanobacter thiooxydans LCS2	Gemmatimonadetes
*J05*	2061979262	3	β-glucosidase	44	YP_002760449.1	Gemmatimonas aurantiaca T-27	Gemmatimonadetes
*J06*	2061979786	1	β-glucosidase	61	ZP_08918778.1	Thermobacillus composti KWC4	Paenibacillus
*J07*	2061980390	1	β-glucosidase	66	NP_242789.1	Bacillus halodurans C-125	Not binned
*J08*	2062002762	1	β-glucosidase	99	YP_003323667.1	Thermobaculum terrenum ATCC BAA-798	Not binned
*J09*	2062002762	1	β-glucosidase	98	YP_003323667.1	Thermobaculum terrenum ATCC BAA-798	Not binned
*J10*	2062002993	3	β-glucosidase	77	ZP_09004353.1	Paenibacillus lactis 154	Not binned
*J11*	2062005533	3	β-glucosidase	42	ZP_06970881.1	Ktedonobacter racemifer DSM 44963	Not binned
*J12*	2062006736	3	β-glucosidase	94	YP_003291338.1	Rhodothermus marinus DSM 4252	Rhodothermus1
*J13*	2062007625	1	β-glucosidase	93	YP_003318753.1	Sphaerobacter thermophilus DSM 20745	Sphaerobacter
*J14*	2062008681	3	β-glucosidase	97	YP_003324065.1	Thermobaculum terrenum ATCC BAA-798	Sphaerobacter
*J15*	2062012385	3	β-glucosidase	75	YP_823953.1	Candidatus Solibacter usitatus Ellin6076	Not binned
*J16*	2062018481	3	β-glucosidase	100	YP_004824792.1	Rhodothermus marinus SG0.5JP17-172	Rhodothermus1
*J17*	2062019328	3	β-glucosidase	71	ZP_08918857.1	Thermobacillus composti KWC4	Paenibacillus
*J18*	2062019735	1	β-glucosidase	99	AAN05441.1	Thermus sp. IB-21	Thermus
*J19*	2062026722	1	β-glucosidase	72	YP_002522957.1	Thermomicrobium roseum DSM 5159	Thermomicrobium
*J21*	2061975668	9	Endoglucanase	54	YP_002759529.1	Gemmatimonas aurantiaca T-27	Gemmatimonadetes
*J22*	2061976479	8	Endoglucanase	72	BAF49077.1	Paenibacillus sp. W-61	Paenibacillus
*J23*	2061977143	5	Endoglucanase	32	ZP_09216417.1	Gordonia amarae NBRC 15530	Sphaerobacter2
*J24*	2061979932	9	Endoglucanase	54	ACJ68032.1	Paenibacillus provencensis	Paenibacillus
*J25*	2061986269	12	Endoglucanase	98	YP_004824941.1	Rhodothermus marinus SG0.5JP17-172	Rhodothermus2
*J26*	2061990001	12	Endoglucanase	100	YP_004824941.1	Rhodothermus marinus SG0.5JP17-172	Not binned
*J27*	2061990054	5	Endoglucanase	35	ZP_09309733.1	Rhodococcus pyridinivorans AK37	Sphaerobacter2
*J28*	2061994288	5	Endoglucanase	98	YP_003323917.1	Thermobaculum terrenum ATCC BAA-798	Sphaerobacter
*J29*	2062006179	5	Endoglucanase	52	BAJ22272.1	Paenibacillus sp. KSM-N546	Paenibacillus
*J30*	2062016312	9	Endoglucanase	54	ZP_08919343.1	Thermobacillus composti KWC4	Not binned
*J31*	2062017860	5	Endoglucanase	57	ZP_08873206.1	Verminephrobacter aporrectodeae	Not binned
*J32*	2062025020	5	Endoglucanase	96	YP_003320228.1	Sphaerobacter thermophilus DSM 20745	Not binned
*J33*	2062027867	8	Endoglucanase	72	ZP_04851456.1	Paenibacillus sp. oral taxon 786 str. D14	Not binned
*J34*	2062029826	6	Endoglucanase	37	ZP_06416445.1	Frankia sp. EUN1f	Thermobaculum
*J35*	2062032441	5	Endoglucanase	35	ZP_08873206.1	Verminephrobacter aporrectodeae	Not Binned
*J36*	2062035244	5	Endoglucanase	100	YP_004823815.1	Rhodothermus marinus SG0.5JP17-172	Rhodothermus1
*J38*	2062019306	3	Cellobiohydrolase	57	ZP_08918880.1	Thermobacillus composti KWC4	Paenibacillus
*J39*	2062019306	3	Cellobiohydrolase	76	ZP_08918880.1	Thermobacillus composti KWC4	Paenibacillus

*Metagenomic bin indicates the predicted source organism. Refer to D’Haeseleer *et al*. for details [19]. Gene sequence and annotation can be found at the Joint Genome Institute’s img/m website (http://img.jgi.doe.gov/cgi-bin/m/main.cgi) under the “Find Genes” tab using the IMG/M gene ID in the table. J20 and J37 were not included in this study.

### Cell-free and E. coli expression and screening of predicted cellulase genes

Each of the 37 predicted metagenome-derived cellulase genes were synthesized and cloned into a custom vector for *in vitro* cell-free expression using a T7 promoter/terminator-based system [20]. Each gene was expressed *in vitro* and screened for Endo, CBH and BG activity (Table 2). For comparison to the cell-free system, each gene was then cloned into the pDEST17 vector for expression in *E. coli* and screened for the same activities (Table 2). There was a large degree of overlap in terms of active genes detected between the two expression methods, but the *E. coli*-based screen detected activity from a larger subset of genes than the cell-free screen (26 versus 19). BG activity was detected for 15 of the 19 predicted BGs, and none of these enzymes showed Endo activity, consistent with their annotation assigned by the JGI and D’haeseleer *et al*. [19]. Furthermore, 12 of these 15 positive candidates exhibited CBH activity on, indicating that these enzymes have activity on glucose oligomers with n >2. For the predicted Endos, activity was detected for 11 of the 16 candidates. In addition to Endo activity, 7 of the 11 Endos also had BG and/or CBH activity. No activity was detected for the two predicted CBH genes.

**Table 2:** Screen of predicted glycoside hydrolase enzymes for β-glucosidase, endoglucanase, and cellobiohydrolase activity

**Gene ID**	**1**	**2**	**3**	**4**	**5**	**6**	**7**	**8**	**9**	**10**	**11**	**12**	**13**	**14**	**15**	**16**	**17**	**18**	**19**	**21**	**22**	**23**	**24**	**25**	**26**	**27**	**28**	**29**	**30**	**31**	**32**	**33**	**34**	**35**	**36**	**38**	**39**	
Endo																							**+**				**+**	**+**	**+**						**+**			Cell-free
																							**+**	**+**	**+**	**+**	**+**	**+**	**+**	**+**	**+**			**+**	**+**			*In vivo*
CBH		**+**	**+**			**+**		**+**	**+**		**+**			**+**	**+**		**+**	**+**	**+**																			Cell-free
	**+**					**+**	**+**	**+**	**+**					**+**			**+**	**+**	**+**				**+**		**+**			**+**	**+**						**+**			*In vivo*
βG	**+**	**+**	**+**			**+**		**+**	**+**		**+**			**+**	**+**		**+**	**+**	**+**											**+**				**+**				Cell-free
	**+**	**+**	**+**		**+**	**+**	**+**	**+**	**+**		**+**			**+**	**+**	**+**	**+**	**+**	**+**						**+**					**+**								*In vivo*

Cell-free and *in vivo* expressed enzymes are labeled in the far right column. Enzyme activities are as follows: Endoglucanase (Endo), cellobiohydrolase (CBH), and β-glucosidase (βG). Detection of enzymatic activity is indicated with a **+** for positive and a blank cell for negative.

### Activity profile of cellulases

Of the 37 enzymes in the initial screen, 15 of the 19 BGs and 6 of the 16 Endos were expressed at sufficient quantities to profile in greater detail. The activity of each enzyme was measured at temperatures ranging from 45 to 99°C, pH between 4.0 and 8.0, and IL concentrations ranging from 0 to 40% [C2mim][OAc] (v/v). These data were then plotted and optimal temperature/pH and IL-tolerance was determined for each enzyme (Table 3). To illustrate the dynamic activity range of each enzyme, the temperature, pH and IL concentration ranges that gave greater than 80 or 50% activity compared to the optimal activity are also reported in Table 3. All of the enzymes were active at elevated temperature, but the range of optimum temperatures (*T*_*opt*_) was broad, ranging from 45 to 95°C. The enzymes were divided into two groups: seven enzymes with a *T*_*opt*_ within 5° of 70°C and another seven near 90°C. Of the remaining enzymes, five had a *T*_*opt*_ below 70°C and two had an intermediate *T*_*opt*_ of 80°C. The enzymes also showed a similar clustering around optimal pH values (*pH*_*opt*_), with fourteen enzymes having a slightly acidic *pH*_*opt*_ between 5.0 and 6.0 and the remaining seven enzymes having a *pH*_*opt*_ between 6.5 and 7.5. However, many of these enzymes were active over a broad pH range, and all but J16 retained ≥50% activity at pH 7.0. Five of the enzymes were more than 80% active at the highest pH tested of 8.0, indicating that these enzymes also tolerate slightly alkaline conditions.

**Table 3:** Activity profile of the active glycoside hydrolase enzymes

**Gene ID**	**01**	**02**	**03**	**05**	**06**	**07**	**08**	**09**	**11**	**14**	**15**	**16**	**17**	**18**	**19**	**24**	**25**	**26**	**29**	**30**	**36**
***T***_***opt ***_**(°C)**	45	90	75	70	65	70	90	90	60	70	70	80	60	95	80	55	95	95	65	50	95
**Temp (°C) ≥80% activity**	45–50	80–90	75	60–75	55–70	65–70	80–90	80–90	55–60	60–75	65–70	75–80	55–60	85–95	70–85	50–60	80–95	85–95	55–70	45–55	75–95
**Temp (°C) ≥50% activity**	45–55	70–90	70–80	45–80	45–75	60–75	65–90	65–90	45–65	50–75	60–70	70–80	45–60	70–95	60–85	45–65	60–95	75–95	50–70	45–55	55–95
***pH***_***opt***_	6	7	5.5	7	6	6	5	5	6	6	6.5	5	6	6	5.5	6.5	7.5	7.5	7.5	6	6
**pH ≥80% activity**	6–6.5	5.5–8	4.5–6	6–7.5	5–6.5	6–7.5	4–8	4–8	5–6.5	5.5–7.5	6–7	5–5.5	5 –7	4.5–7	4.5–7.5	5.5–7.5	4–8	5.5–8	5.5–8	5–7	6–7.5
**pH ≥50% activity**	5–7	4.5–8	4.5–7	5.5–8	4.5–7.5	5.5–8	4–8	4–8	4.5–7	5–8	6–7.5	5–6.5	5–7.5	4–8	4.5–8	5–8	4–8	4–8	5–8	4.5–8	5.5–8
**IL% (v/v) ≥100% activity**	0	15	40	35	5	10	0	0	10	5	0	35	0	5	10	10	40	30	5	25	35
**IL% (v/v) ≥80% activity**	0	25	40	40	5	15	5	5	15	10	0	40	0	15	20	10	40	35	10	30	35
**IL% (v/v) ≥50% activity**	15	35	40	40	5	15	20	20	20	20	0	40	5	30	30	15	40	35	15	35	35
Max activity in IL*	0.68 (5)	1.1 (5)	1.2 (40)	2.1 (15)	1.3 (5)	1.2 (5)	0.87 (5)	0.89 (5)	1.5 (5)	1.2 (5)	0.45 (5)	5 (10)	0.5 (5)	1.2 (5)	1.1 (5)	2.1 (5)	1.9 (15)	2.5 (15)	1.4 (5)	2.5 (15)	2 (25)

Enzyme activity was profiled at temperatures between 45 and 95°C, pH between 4 and 8, and IL concentrations between 0 and 40% (v/v) of [C_2_mim][OAc]. The temperature and pH that elicited the highest activity is indicated in row T_opt_ and pH_opt_, respectively. Temperature and pH ranges that permitted greater than 80% and 50% activity are indicated below the optimum value. IL-tolerance is indicated as the maximum concentration of [C_2_mim][OAc] that permits at least 80% and 50% enzyme activity (i.e. a value of 15 in the 80% row would indicate that 15% (v/v) of [C_2_mim][OAc] is the maximum concentration of [C_2_mim][OAc] that can be used to retain at least 80% enzyme activity). Most enzymes showed a steady decline in activity with increasing IL concentrations. *Max activity in IL is reported as the highest fold change of activity in the presence of IL compared to water and the () indicates the IL concentration (v/v) in which that highest activity as achieved. Values less than 1 indicate the enzyme is less active in IL than in water while values greater than 1 indicate the enzyme has increased activity in the presence of IL.

Enzyme activity was profiled at temperatures (Temp) between 45 and 95°C, pH between 4 and 8, and IL concentrations between 0 and 40% (v/v) of 1-ethyl-3 methylimidazolium acetate [C2mim][OAc]. The temperature and pH that elicited the highest activity is indicated in the row for optimum temperature (*T*_*opt*_) and optimal PH values (*pH*_*opt*_), respectively. Temperature and pH ranges that permitted greater than 80% and 50% activity are indicated below the optimum value. Ionic liquid (IL)-tolerance is indicated as the maximum concentration of [C2mim][OAc] that permits at least 80% and 50% enzyme activity (that is, a value of 15 in the 80% row would indicate that 15% (v/v) of [C2mim][OAc] is the maximum concentration of [C2mim][OAc] that can be used to retain at least 80% enzyme activity). Most enzymes showed a steady decline in activity with increasing IL concentrations. *Maximum (Max) activity in IL is reported as the highest fold change of activity in the presence of IL compared to water and the values in brackets are the IL concentrations (v/v) in which that highest activity was achieved. Values less than 1 indicate the enzyme is less active in IL than in water, and values greater than 1 indicate the enzyme had increased activity in the presence of IL.

**Figure 1:**
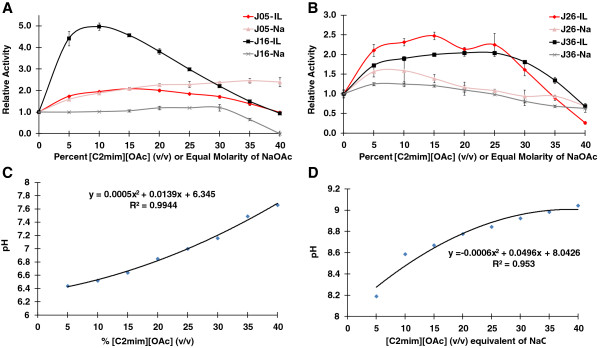
**Plot of enzyme activity in the presence of 0 to 40% 1-ethyl-3 methylimidazolium acetate [C2mim][OAc] or an equal molarity of sodium acetate (NaOAc).** Relative activity is based on activity in water (0% ionic liquid (IL) value). **(A)** Two IL-tolerant β-glucosidases and **(B)** two IL-tolerant endoglucanases were profiled. The pH was determined at each concentration of **(C)** [C2mim][OAc] and **(D)** NaOAc. Error bars represent one standard deviation (they are too small to be visualized on **C** and **D**).

Surprisingly, most of the enzymes (16 of the 21 tested) showed an initial increase in activity in the presence of [C2mim][OAc] compared to water (0% IL), with a 15 to 500% enhancement in activity that eventually declined at higher [C2mim][OAc] concentrations (Table 3). This phenomenon is illustrated in the row labeled “Max activity in IL” in Table 3 that lists the highest fold change in activity in the presence of [C2mim][OAc]. For example, enzyme J16 was found to be five times more active in 10% (v/v) [C2mim][OAc] than in water. The majority of the enzymes were active in at least 20% (v/v) [C2mim][OAc] and maintained greater than 50% activity. Six of the enzymes (J03, J05, J16, J25, J26 and J36) maintained more than 80% activity in 35 to 40% [C2mim][OAc]. Only a single enzyme, J15, lost activity at low [C2mim][OAc] concentrations. The BG enzymes J5 and J16 and Endo enzymes J26 and J36 showed the highest increase in activity in the presence of [C2mim][OAc]. To examine the relationship of IL-tolerance to potential halo-tolerance, their activity was measured in equal molar concentrations of [C2mim][OAc] and sodium acetate (NaOAc) (Figure 1A-B). Each of these enzymes also showed greater or equal activity in the presence of NaOAc, despite this salt buffering the solution at a more basic pH, which tends to be outside the optimal activity range for these enzymes (in water), especially J16 (Figure 1C-D).

**Figure 2:**
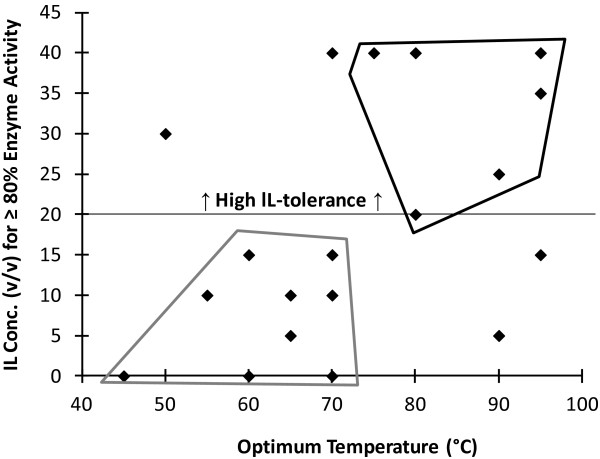
**A plot highlighting the correlation between thermotolerance and ionic liquid (IL)-tolerance of the enzymes shown in Table**3**.** The plot shows the maximum [C2mim][OAc] concentration that permits ≥80% enzyme activity compared to water versus the optimum temperature (*T*_*opt*_) of the enzyme. There are two overlapping data points at 95°C, 35% IL. Enzymes with high IL-tolerance are defined as the enzymes that can tolerate 20% (v/v) [C2mim][OAc] or greater (above horizontal line). The enzymes fall into two clusters: the black polygon where 78% (7/9) of the enzymes with a *T*_*opt*_ >70°C have high IL-tolerance, and the grey polygon where 82% (9/11) of the enzymes with a *T*_*opt*_ ≤70°C have low or no IL-tolerance. Only 18% (2/11) of the enzymes with a *T*_*opt*_ ≤70°C have high IL-tolerance.

The *T*_*opt*_ and *pH*_*opt*_ values of these enzymes were compared to their IL-tolerance to determine whether either of these properties positively correlates with high IL-tolerance. A plot of the optimum temperature or pH of the enzyme versus the highest concentration of [C2mim][OAc] in which the enzyme retains ≥80% of its activity was examined for clustering of values that would indicate that a particular range of pH or temperature positively correlates with high IL-tolerance. Of these two properties, only the *T*_*opt*_ showed any discernible correlation with high IL-tolerance (Figure 2). It appears that a *T*_*opt*_ >70°C is a positive indicator of high IL-tolerance. Enzymes with a *T*_*opt*_ ≤70°C have only an 18% probability of being highly tolerant to [C2mim][OAc], whereas enzymes with a *T*_*opt*_ >70°C have a 78% chance of being highly IL-tolerant (see Figure 2 legend for details).

## Discussion

Developing IL-tolerant enzymatic mixtures for cellulose hydrolysis will permit the advancement of technologies that combine IL-based pretreatment using [C2mim][OAc] with enzymatic hydrolysis. This type of process intensification will be critical for the development of cost-competitive lignocellulosic biofuel technologies [11]. However, there are few IL-tolerant enzymes known and more must be discovered before these technologies can be matured to the point of large-scale implementation in a biorefinery. This study was based on the hypothesis that thermotolerance and IL-tolerance are correlated, and therefore sought to expand the list of known IL-tolerant enzymes by identifying, expressing, and characterizing multiple thermophilic biomass deconstructing enzymes sourced from a single compost-derived microbial community that was previously used as a test bed for comparing IL and thermotolerance [14,19]. In the course of this study, we compared cell-free and *in vivo E. coli* expression methods for rapidly (and with high fidelity) screening through predicted enzyme candidates to narrow down the list of targets to functional and properly annotated enzymes. Results from this study elicit several interesting conclusions regarding the utility of *in vitro* versus *in vivo* screening methods, the activity of the recombinant enzymes versus the native enzymes from the parent microbial community, and the hypothesis that thermotolerance and IL-tolerance are correlated.

Comparison of the cell-free and *in vivo E. coli* screens yielded several observations: 1) both screens work well at quickly screening through candidate genes to identify functional genes; 2) the screens produce similar results in regards to predicted annotation and 3) the cell-free screen is more rapid (24 hours) compared to the *in vivo* screen (5 days); however, 4) the cell-free screen missed about 27% of the positive candidates (19 versus 26), and 5) the cell-free screen will eventually require porting into an *in vivo* expression system to conduct more detailed enzyme profiling. In light of these observations, the cell-free screen would be advantageous if the number of candidates to screen is large, as it is more rapid and less labor-intensive than the *in vivo* screen, whereas the *in vivo* screen would be more advantageous in smaller screens as it provides greater returns and enables more detailed characterization. Overall, the assigned annotation of each enzyme accurately reflected its measured activity. Several enzymes showed activity on multiple substrates, but in most cases the highest measured activity matched the annotation of the enzyme.

After the initial screening, there were 21 promising enzyme targets (15 BG and 6 Endo) to profile in more detail for optimum temperature, pH and IL-tolerance. The profiles revealed that the enzymes are indeed thermotolerant (*T*_*opt*_ between 45 and 95°C), and the two clusters of optimum temperatures observed for these enzymes (70 and 90°C) mirror the pattern seen in the profile of the native enzymes produced by the parent community from which these genes were isolated, except that the native enzymes had their had two *T*_*opt*_ peaks 10° lower than the heterologous enzymes (60 and 80°C) [14]. It is unclear why this may be. Perhaps the community produces a complex mixture of enzymes, the average of which results in observed *T*_*opt*_ at around 60 and 80°C, or the community only expresses a complement of enzymes with *T*_*opt*_ near 60 and 80°C.

The native enzymes produced by the parent microbial community were also [C2mim][OAc]-tolerant, a trait mirrored by the majority of enzymes profiled in this study. However, unlike the recombinant enzymes in this study, the native cellulase enzymes were not observed to have an increase in activity in the presence of ILs [14]. Many of the enzymes in this study showed an increase in activity in the lower range of [C2mim][OAc] concentrations tested (0.3 to 0.9 M), some several fold higher than the activity in water. The fact that several of these enzymes also showed increased activity in the presence of NaOAc suggests that these enzymes may require the presence of salt for optimal activity. The increase in activity with NaOAc was not as high for enzyme J16 as in the corresponding concentration of IL, which is likely due to the more basic pH of NaOAc and the lower pH optimum of J16 (pH 5.0). This phenomenon was less apparent for the other enzymes tested, but generally the enzymes demonstrated relatively higher levels of activity in the presence of [C2mim][OAc] compared to NaOAc. This apparent IL- and salt-tolerance is not surprising, considering that these enzymes are similar to those derived from thermotolerant and slightly halo-tolerant organisms like *Rhodothermus marinus*, which requires salt and grows optimally in about 0.3 M NaCl [21]. Unlike many fungal enzymes, these cellulases tend to prefer more neutral pH (6.0 or 7.0), and many retained more than 80% activity at the highest pH tested of 8.0. [C2mim][OAc] buffers around neutral pH in the range of concentrations tested, a property that may further ameliorate tolerance to this IL by several of the enzymes tested. The affinity of these enzymes for more neutral pH may reflect their origin; for example, *R. marinus* grows optimally at pH 7.0 [21].

The mechanisms of IL-tolerance are not well understood; few enzymes have been investigated for IL-tolerance in general and there are no studies that have looked at a large enough set of enzymes with a single type of IL, such as [C2mim][OAc], to do any type of thorough comparative analysis. The 21 enzymes characterized in this study had varying degrees of [C2mim][OAc]-tolerance and therefore provide an opportunity to look for correlations between IL-tolerance and other characteristics of the enzymes, that is, *T*_*opt*_ and pH ranges. Of those two properties, there only appears to be a correlation between IL-tolerance and *T*_*opt*_, consistent with the conclusion from studies of other thermotolerant enzymes. A comparison of the IL-tolerance and *T*_*opt*_ revealed that the enzymes with a *T*_*opt*_ >70°C tend to have a higher probability of tolerating high concentrations of [C2mim][OAc]. This indicates that evolution towards higher *T*_*opt*_ frequently alters the properties of an enzyme in a manner that also promotes tolerance to [C2mim][OAc]. The data from this study also indicates that the correlation is not simply between thermotolerance and IL-tolerance but more specifically hyperthermotolerance and IL-tolerance. Only a single enzyme studied with a *T*_*opt*_ <70°C displayed appreciable levels of IL-tolerance. This observation helps explain why enzymes from filamentous fungi used in commercial cellulase cocktails do not display IL-tolerance; there are no known hyperthermophilic filamentous fungi. Furthermore, future studies aimed at studying the mechanisms of [C2mim][OAc]-tolerance may benefit from a refined hypothesis that hyperthermotolerance (>70°C) is correlated with IL-tolerance.

The results presented here can also be used to comment on the general strategy used to identify enzymes with a particular set of characteristics, in this case IL-tolerance. The microbial community from which these enzymes were derived was originally established under the premise that organisms endowed with a particular functionality could be selectively enriched in abundance from a complex microbial community by cultivation under defined conditions. This selective enrichment could then help researchers target organisms and genes with a desired set of characteristics. In this case, the desired functionality was production of cellulase enzymes and the desired characteristic was thermo- and IL-tolerant cellulase enzymes. This strategy was implemented by cultivating a microbial community derived from green-waste compost under thermophilic conditions with plant biomass as a sole carbon source [14]. The native enzymes produced by this community were both thermo- and IL-tolerant and so were the recombinant enzymes derived from this community, suggesting that selective cultivation is a good method for discovering enzymes that function under a desired set of conditions.

## Conclusions

The enzymes characterized in this report are some of the most tolerant to [C2mim][OAc] reported to date [12,14,15,17]. Tolerance to this particular IL is of increasing interest as it is currently one of the most effective and well-studied ILs for pretreatment of lignocellulosic biomass [22]. Recent efforts to develop IL-tolerant cellulase cocktails and to incorporate these cocktails into one-pot pretreatment and saccharification bioprocessing schemes show that IL-tolerant enzymes can be used to develop new technologies to deconstruct biomass, and open up the technological landscape for lignocellulosic biorefineries [18]. The enzymes described in this report can be used to further those technologies.

## Methods

### Manual cellulase gene assembly

Although most of the full-length ORFs in Table 1 were taken directly from the metagenome, several were manually reconstructed from fragmented genes identified in the assembly of the metagenomic dataset. The following ORFs were manually assembled: J03 had an incorrectly predicted start codon. The start of this ORF was moved 5’ to match the start of its top BLAST hit. J08/09 are two versions of a single ORF composed of four gene fragments from the metagenome (IMG gene IDs 2061981261, 2062002762, 2062037967, 2061992858), which all have very high homology with a predicted BG from *Thermobaculum terrenum* ATCC BAA-798 [GenBank: ACZ42845.1]. J08 is an assembly of 2061981261 (N-terminus), 2062002762 (C-terminus), and ACZ42845.1 (sequence that encodes AAIVITENGAAYPDE inserted between the two sequences), and J09 is a compilation of 2062037967, 2061992858, and the same fragment from ACZ42845.1 assembled in the same order as J08. Overall, J08 and J09 differ by 5 AA. The same situation applies to J10, which is assembled from 2062002992 (N-terminus), 2062002993 (C-terminus), and a middle fragment (sequence encoding NAVKVTAAA) from ACX65411.1, a glycoside hydrolase family 3 protein from *Geobacillus sp.* Y412MC1. J11 was also assembled in the same manner; two consecutive ORFs (2062005533 and 2062005534) were merged with a fragment encoding (YVR) derived from a glycoside hydrolase family 3 protein from *Ktedonobacter racemifer* DSM 44963 (EFH83601.1). J38/39 are two versions of two consecutive orfs (2062019305, and 2062019306), which may be separated by a single base pair frame-shift or a larger deletion. J38 is a merger of the two orfs by inserting a single base pair to encode a leucine codon at residue 103. J39 is a merger of the two ORFs with a 316 base pair insertion at the same location derived from a BG from *Paenibacillus sp.* JDR-2 (ACT00588.1), to repair the glycoside hydrolase family 3 N-terminal domain.

### Gene synthesis and cloning

Each gene was codon-optimized for expression in *E. coli* and synthesized by Genscript (Piscataway, NJ, USA). They were then cloned into a modified pUC57 vector constructed at Genscript, pUC57CFv1, with an added T7 promoter and terminator, as well as gateway attB1/attB2 sequences flanking the ORF, and a 8 × C-terminal 8 × His and Strep-tag II dual tag. There was an in-frame NheI-XhoI cloning site added between the attB1/attB2 sequences to place the ORFs into the pUC57CFv1 vector. The added vector sequences were cloned into the pUC57 vector at the EcoRI and SacI sites. Synthesized ORFs were then cloned into the pUC57CFv1 vector at the NheI-XhoI sites. The synthesized genes in the pUC57CFE1 vector were transformed in to TOP10 *E. coli* for storage at -80°C.

The T7, Gateway attB1/attB2 and His tag sequences added to pUC57 are:

GAATTCTAAATTAATACGACTCACTATAGGGAGACCACAACGGTTTCCCTCTAGAAATAATTTTGTT TAACTTTAAGAAGGAGATATACATATGACAAGTTTGTACAAAAAAGCAGGCTTCGCTAGCCCAATCC AATCTCGAGGACCCAGCTTTCTTGTACAAAGTGGTCCATCATCACCATCACCATTAACAATAACTAG CATAACCCCTTGGGGCCTCTAAACGGGTCTTGAGGGGTTTTTTGGAGCTC

#### ***In vitro and in vivo expression of cellulases***

Each of the 37 cellulases was expressed *in vitro* using the RTS 100 *E. coli* 100 Hy cell-free expression Kit (Roche Diagnostics, Mannheim, Germany, Catalogue Number 03 186 148 001), using 0.5 μg of vector and following the manufacturer's instructions. The lyophilized plasmids were dissolved in DNase/RNase-free water before use. The *in vitro* protein expression was performed at 30°C for six hours. The expression products were used immediately for enzyme assay reactions.

To validate the enzyme activity results of *in vitro* protein expression and assays, the cellulase genes were cloned into the low-copy bacterial expression plasmid pDEST17 by Gateway cloning techniques following the manufacturer's instructions (Invitrogen). The sequences of all cloned genes in the pDONR221 and pDEST17 vectors were verified by DNA sequencing (Quintara Biosciences; Albany, CA, USA). All cellulase genes in the pDEST17 vector, except J24 and J29, were transformed into BL21(DE3)Star *E. coli* (Invitrogen, Carlsbad, CA, USA). The J24 and and J29 genes in the pDEST17 vector were transformed into the T7 Express I^q^*E. coli* strain (New England BioLabs, Ipswich, Massachusetts, USA) to attenuate the basal level of cellulase expression during the growth phase prior to induction of protein expression. This was done because the expression vectors containing J24 and J29 were toxic to TOP10 and BL21(DE3)Star strains of *E. coli*, presumably due to the leaky activation of the T7 promoter. Bacterial cultures were grown in 96-deep well-plates in 800 μL of Luria-Bertani (LB) Miller broth containing carbenicillin (50 μg/ml) in each well. The overnight cultures of *E. coli* were inoculated to fresh LB medium containing Overnight Express Autoduction System 1 (Calbiochem, San Diego, CA, USA) reagent and carbenicillin. In the autoinduction medium, the bacterial cultures were incubated at 37°C with constant shaking at 200 rpm for the first four hours. Then the cultures were grown at 30°C for 18 hours with constant shaking at 200 rpm. The cell pellets were harvested by centrifugation at 6,000 g for 30 minutes, and then stored at -20°C. Each of the frozen cell pellets was thawed and resuspended in 0.1 mL of BugBuster containing lysozyme (1 mg/mL), benzonase (25 U/ml) and phenylmethanesulfonylfluoride (PMSF) (1 mM). After 30 minutes of incubation at room temperature, the cell lysates were centrifuged at 4,000 g for 30 minutes at 4°C. The soluble protein extracts (supernatants) were filtered through 0.45-μm syringe filters, and then used for enzymatic assays.

### Enzyme assays for *in vitro* and *in vivo* screens

The enzyme activities of the *in vitro* protein expression products from the pUC57CFE1 vector were screened on the following substrates: 4-nitrophenyl-β-D-glucopyranoside (*p*NPG, 5 mM), 4-nitrophenyl-β-D-cellobioside (*p*NPC, 5 mM), and 1% carboxymethyl cellulose (Sigma Aldrich). Each enzyme reaction mixture containing one of these substrates and 5 μL of *in vitro* expression product or soluble extract from *E. coli* cell lysates (before or after induction) was done in 50 mM sodium acetate buffer at pH 5 in a total volume of 50 μL. The final concentration of 4-nitrophenol-labeled substrate (*p*NPC, or *p*NPG) was 5 mM, and that of carboxymethylcellulose (CMC) was 1% in each reaction. The enzymatic reaction was done at 50°C for 16 hours. For the reaction mixtures containing CMC, a 3,5-dinitrosalicylic acid (DNS) assay was used to quantify hydrolyzed products. For the reaction mixtures containing *p*NPG, or *p*NPC, an equal volume of 2% sodium carbonate (Na_2_CO_3_) was added prior to measuring absorbance at 420 nm to detect hydrolyzed 4-nitrophenol.

### Enzyme assays for activity profiling of cellulases

To profile the enzyme activity of positive cellulases in the screen, each enzyme was expressed *in vivo* as described above, except the culture volume was scaled to 50 ml. For each enzyme assay, 5 to 20 ul of lysate was used to ensure that each enzyme had an activity that fell within the linear range of the activity assay. Enzymes J1 to J19 were screened using *p*NPG (5 mM final concentration) and enzymes J21 to J39 were screened using CMC (1% w/v final concentration) in a 100-ul reaction volume. Each value reported in Table 3 is from the average of triplicate reactions. For the temperature profile, the reaction was set up using 50 mM 2-(N-morpholino)ethanesulfonic acid (MES) buffer pH 6.5, and reactions were run for 15 to 60 minutes, depending on enzyme activity, at 5° increments from 45 to 99°C. For the pH profile, the reactions were run at approximately 10°C below the optimal temperature of each enzyme in 100 mM NaOAc 50 mM MES and 50 mM 4-(2-hydroxyethyl)-1-piperazineethanesulfonic acid (HEPES) buffers between pH 4.0 and 8.0. The buffers were made by mixing two aliquots of the aforementioned buffer set to either pH 4.0 (buffer A) or 8.0 (buffer B) in 10% increments, starting from 0% B to 100% B, giving 11 points total between pH 4.0 and 8.0. For IL-tolerance profiles, the reactions were run without added buffer in IL concentrations between 0 and 40% w/v [C2mim][OAc] at approximately 10°C below the optimal temperature of each enzyme. Reaction times were set to keep the values within the linear range of detection. For some enzymes, the same reaction was set up substituting an equal molar amount of NaOAc for [C2mim][OAc]. Figure 1C-D shows the pH at each concentration of IL and molar equivalent concentrations of NaOAc.

## Abbreviations

BG: β-glucosidase; [C2mim][OAc]: 1-ethyl-3 methylimidazolium acetate; CBH: cellobiohydrolase; Endo: Endoglucanase; GH: Glycoside hydrolase; HEPES: 4-(2-hydroxyethyl)-1-piperazineethanesulfonic acid; IL: Ionic liquid; MES: 2-(N-morpholino)ethanesulfonic acid; NaOAC: Sodium acetate; ORF: Open reading frame; PMSF: phenylmethanesulfonylfluoride; pNPC: 4-nitrophenyl-β-D-cellobioside; pNPG: 4-nitrophenyl-β-D-glucopyranoside; Na2CO3: Sodium carbonate; Topt: Optimum temperature.

## Competing interests

The authors declare that they have no competing interests.

## Authors’ contributions

JMG carried out most of the research and wrote the manuscript. JMG and JIP designed the study, designed the *in vitro* expression vector and cloned all cellulase genes to *E. coli* expression vector, and conducted the *in vitro* and *in vivo* screens. JB and VR profiled enzyme activities. PD identified and repaired the 37 cellulase ORFs. BFQ advised JB. KLS advised JIP and VR and helped visualize *T*_*opt*_ and IL-tolerance. SWS and BAS advised JG. All authors read, provided edits, and approved the manuscript.

## Supplementary Material

Additional file 1 **Table giving detailed annotation information on predicted cellulases.** Shown are predicted cellulase genes investigated in this report, detailing additional annotations for each gene, including E.C. number and glycoside family predictions. Click here for file

## References

[B1] Laboratory ORN U.S. Billion-Ton Update: Biomass Supply for a Bioenergy and Bioproducts Industry. US DOE Energy Efficiency and Renewable Energy web site 2011 http://www1.eere.energy.gov/bioenergy/pdfs/billion_ton_update.pdf

[B2] Wiselogel AE Agblevor FA Johnson DK Deutch S Fennell JA Sanderson MA Compositional changes during storage of large round switchgrass bales Bioresource Technol 1996 56 103 109 10.1016/0960-8524(95)00171-9

[B3] Wald ML U.S. Backs Project to Produce Fuel From Corn Waste The New York Times, New York edition July 7th 2011 B10

[B4] U.S. DOE Using Fermentation and Catalysis to Make Fuels and Products: BIOCHEMICAL CONVERSION. US DOE Energy Efficiency and Renewable Energy web site 2010 http://www1.eere.energy.gov/bioenergy/pdfs/biochemical_four_pager.pdf

[B5] Steen EJ Kang Y Bokinsky G Hu Z Schirmer A McClure A Del Cardayre SB Keasling JD Microbial production of fatty-acid-derived fuels and chemicals from plant biomass Nature 2010 463 559 562 10.1038/nature08721 20111002

[B6] Peralta-Yahya PP Keasling JD Advanced biofuel production in microbes Biotechnol J 2010 5 147 162 10.1002/biot.200900220 20084640

[B7] Nakayama S Kiyoshi K Kadokura T Nakazato A Butanol production from crystalline cellulose by cocultured clostridium thermocellum and clostridium saccharoperbutylacetonicum N1-4 Appl Environ Microb 2011 77 6470 6475 10.1128/AEM.00706-11 PMC3187149 21764954

[B8] Li C Knierim B Manisseri C Arora R Scheller HV Auer M Vogel KP Simmons BA Singh S Comparison of dilute acid and ionic liquid pretreatment of switchgrass: biomass recalcitrance, delignification and enzymatic saccharification Bioresource Technol 2010 101 4900 4906 10.1016/j.biortech.2009.10.066 19945861

[B9] Zhao H Jones CIL Baker GA Xia S Olubajo O Person VN Regenerating cellulose from ionic liquids for an accelerated enzymatic hydrolysis J Biotechnol 2009 139 47 54 10.1016/j.jbiotec.2008.08.009 18822323

[B10] Tadesse H Luque R Advances on biomass pretreatment using ionic liquids: An overview Energ Environ Sci 2011 4 3913 3929 10.1039/c0ee00667j

[B11] Klein-Marcuschamer D Simmons BA Blanch HW Techno-economic analysis of a lignocellulosic ethanol biorefinery with ionic liquid pre-treatment Biofuels Bioprod Biorefin 2011 5 562 569 10.1002/bbb.303

[B12] Turner MB Spear SK Huddleston JG Holbrey JD Rogers RD Ionic liquid salt-induced inactivation and unfolding of cellulase from Trichoderma reesei Green Chem 2003 5 443 447 10.1039/b302570e

[B13] Park JI Steen EJ Burd H Evans SS Redding-Johnson AM Batth T Benke PI D'Haeseleer P Sun N Sale KL Keasling JD Lee TS Petzold CJ Mukhopadhyay A Singer SW Simmons BA Gladden JM A thermophilic ionic liquid-tolerant cellulase cocktail for the production of cellulosic biofuels PLoS One 2012 7 e37010 10.1371/journal.pone.0037010 22649505 PMC3359315

[B14] Gladden JM Allgaier M Miller CS Hazen TC VanderGheynst JS Hugenholtz P Simmons BA Singer SW Glycoside hydrolase activities of thermophilic bacterial consortia adapted to switchgrass Appl Environ Microbiol 2011 77 5804 5812 10.1128/AEM.00032-11 21724886 PMC3165268

[B15] Datta S Holmes B Park JI Chen ZW Dibble DC Hadi M Blanch HW Simmons BA Sapra R Ionic liquid tolerant hyperthermophilic cellulases for biomass pretreatment and hydrolysis Green Chem 2010 12 338 345 10.1039/b916564a

[B16] Gladden JM Eichorst SA Hazen TC Simmons BA Singer SW Substrate perturbation alters the glycoside hydrolase activities and community composition of switchgrass-adapted bacterial consortia Biotechnol Bioeng 2012 109 1140 1145 10.1002/bit.24388 22125273

[B17] Zhang T Datta S Eichler J Ivanova N Axen SD Kerfeld CA Chen F Kyrpides N Hugenholtz P Cheng J-F Sale KL Simmons BA Rubin E Identification of a haloalkaliphilic and thermostable cellulase with improved ionic liquid tolerance Green Chem 2011 13 2083 2090 10.1039/c1gc15193b

[B18] Shi J Gladden JM Sathitsuksanoh N Kambam P Sandoval L Mitra D Zhang S George A Singer SW Simmons BA Singh S One-pot ionic liquid pretreatment and saccharification of switchgrass Green Chem 2013 15 2579 2589 10.1039/c3gc40545a

[B19] D'haeseleer P Gladden JM Allgaier M Chain PSG Tringe SG Malfatti SA Aldrich JT Nicora CD Robinson EW Paša-Tolić L Hugenholtz P Simmons BA Singer SW Proteogenomic analysis of a thermophilic bacterial consortium adapted to deconstruct switchgrass PLoS ONE 2013 8 e68465 10.1371/journal.pone.0068465 23894306 PMC3716776

[B20] Rosenberg AH Lade BN Chui DS Lin SW Dunn JJ Studier FW Vectors for selective expression of cloned DNAs by T7 RNA polymerase Gene 1987 56 125 135 10.1016/0378-1119(87)90165-X 3315856

[B21] Bjornsdottir SH Blondal T Hreggvidsson GO Eggertsson G Petursdottir S Hjorleifsdottir S Thorbjarnardottir SH Kristjansson JK Rhodothermus marinus: physiology and molecular biology Extremophiles 2006 10 1 16 10.1007/s00792-005-0466-z 16075163

[B22] Sathitsuksanoh N George A Zhang YHP New lignocellulose pretreatments using cellulose solvents: a review J Chem Technol Biotechnol 2013 88 169 180 10.1002/jctb.3959

