# Supplementary material for: Discovery and characterization of ionic liquid-tolerant thermophilic cellulases from a switchgrass-adapted microbial community
Source: Biotechnol Biofuels. 2014 Jan 29;7:15. doi: 10.1186/1754-6834-7-15 (PMC3923250; doi:10.1186/1754-6834-7-15)
Supplement: Additional file 1 — Table giving detailed annotation information on predicted cellulases. Shown are predicted cellulase genes investigated in this report, detailing additional annotations for each gene, including E.C. number and glycoside family predictions. [file 1754-6834-7-15-S1.docx]

**Additional File 1. Detailed annotation information of predicted cellulases**.

| **Gene ID** | **IMG Scaffold** | **Produc Name** | **EC** | **dbCAN** | **CAZy best BLAST** | **CAZy best EC** |
| --- | --- | --- | --- | --- | --- | --- |
| J01 | contig18198 | Beta-glucosidase-related glycosidases | 3.2.1.21 | GH3 | GH3 | 3.2.1.21 |
| J02 | contig14577 | Beta-glucosidase-related glycosidases | 3.2.1.21 | GH3 | GH3 | 3.2.1.37,3.2.1.55 |
| J03 | contig00241 | Glycosyl hydrolase family 3 C terminal domain. |  |  | GH3 | 3.2.1.21 |
| J04 | contig18211 | beta-galactosidase | 3.2.1.21 | GH1 | GH1 | 3.2.1.21 |
| J05 | contig00260 | Beta-glucosidase-related glycosidases | 3.2.1.21 | GH3 | GH3 | 3.2.1.52 |
| J06 | contig18222 | broad-specificity cellobiase (EC 3.2.1.21) | 3.2.1.21 | GH1 | GH1 | 3.2.1.21 |
| J07 | contig18226 | broad-specificity cellobiase (EC 3.2.1.21) | 3.2.1.21 | GH1 | GH1 | 3.2.1.21 |
| J08 | contig20876 | broad-specificity cellobiase (EC 3.2.1.21) | 3.2.1.21 | GH1 | GH1 | 3.2.1.21 |
| J09 | contig20876 | broad-specificity cellobiase (EC 3.2.1.21) | 3.2.1.21 | GH1 | GH1 | 3.2.1.21 |
| J10 | contig12723 | Beta-glucosidase-related glycosidases |  | GH3 | GH3 | 3.2.1.21 |
| J11 | contig02456 | Glycosyl hydrolase family 3 C terminal domain. |  |  | GH3 | 3.2.1.21 |
| J12 | contig19900 | Beta-glucosidase-related glycosidases |  | GH3 | GH3 | 3.2.1.21 |
| J13 | contig00750 | broad-specificity cellobiase (EC 3.2.1.21) | 3.2.1.21 | GH1 | GH1 | 3.2.1.21 |
| J14 | contig06186 | Beta-glucosidase-related glycosidases | 3.2.1.21 | GH3 | GH3 | 3.2.1.21 |
| J15 | contig03269 | Beta-glucosidase-related glycosidases | 3.2.1.21 | GH3 | GH3 | 3.2.1.37 |
| J16 | contig19490 | Beta-glucosidase-related glycosidases | 3.2.1.21 | GH3 | GH3 | 3.2.1.37 |
| J17 | contig00009 | Beta-glucosidase-related glycosidases | 3.2.1.21 | GH3 | GH3 | 3.2.1.21 |
| J18 | contig18999 | broad-specificity cellobiase (EC 3.2.1.21) | 3.2.1.21 | GH1 | GH1 | 3.2.1.21 |
| J19 | contig18557 | broad-specificity cellobiase (EC 3.2.1.21) | 3.2.1.21 | GH1 | GH1 | 3.2.1.21 |
| J21 | contig18203 | Glycosyl hydrolase family 9. |  | GH9 | GH9 |  |
| J22 | contig18207 | Endoglucanase Y | 3.2.1.4 | GH8 | GH8 | 3.2.1.156 |
| J23 | contig18209 | Cellulase (glycosyl hydrolase family 5). |  | GH39 |  |  |
| J24 | contig18223 | Glycosyl hydrolase family 9./N-terminal ig-like domain of cellulase. | 3.2.1.4 | GH9, CBM30 | GH9,CBM4 | 3.2.1.4 |
| J25 | contig18761 | Glycosyl hydrolase family 12. |  | GH12 | GH12 | 3.2.1.4 |
| J26 | contig18283 | Glycosyl hydrolase family 12. |  | GH12 | GH12 | 3.2.1.4 |
| J27 | contig00348 | Cellulase (glycosyl hydrolase family 5). |  | GH39 | GH39 |  |
| J28 | contig00384 | Endoglucanase | 3.2.1.4 | GH5 | GH5 | 3.2.1.4 |
| J29 | contig00006 | Endoglucanase | 3.2.1.4 | GH5, CBM46 | GH5,CBM46 | 3.2.1.- |
| J30 | contig00568 | Glycosyl hydrolase family 9./N-terminal ig-like domain of cellulase. | 3.2.1.4 | GH9, CBM30 | GH9 | 3.2.1.4 |
| J31 | contig01865 | Endoglucanase |  | GH5 | GH5 |  |
| J32 | contig11864 | Cellulase (glycosyl hydrolase family 5). |  | GH39 | GH39 |  |
| J33 | contig00675 | Endoglucanase Y | 3.2.1.4 | GH8 | GH8 | 3.2.1.156 |
| J34 | contig18583 | Cellobiohydrolase A (1,4-beta-cellobiosidase A) | 3.2.1.4 | GH6 | GH6 | 3.2.1.4 |
| J35 | contig10829 | Endoglucanase |  | GH5 | GH5 |  |
| J36 | contig19677 | Endoglucanase |  | GH5 | GH5 | 3.2.1.4 |
| J38 | contig00009 | Beta-glucosidase-related glycosidases | 3.2.1.21 | CBM6 | GH3,CBM6 | 3.2.1.74 |
| J39 | contig00009 | Beta-glucosidase-related glycosidases | 3.2.1.21 | CBM6 | GH3,CBM6 | 3.2.1.74 |

EC stands for Enzyme Commission. The dbCAN column indicates the glycoside hydrolase family predicted by that database <http://csbl.bmb.uga.edu/dbCAN/>. CAZy best BLAST and EC represent the glycoside hydrolase family and EC from the best BLAST match of the CAZy database <http://www.cazy.org/>.
